# Presepsin (Soluble CD14 Subtype): Reference Ranges of a New Sepsis Marker in Term and Preterm Neonates

**DOI:** 10.1371/journal.pone.0146020

**Published:** 2015-12-31

**Authors:** Lorenza Pugni, Carlo Pietrasanta, Silvano Milani, Claudia Vener, Andrea Ronchi, Mariella Falbo, Milena Arghittu, Fabio Mosca

**Affiliations:** 1 NICU, Department of Clinical Sciences and Community Health, Fondazione IRCCS Ca’ Granda Ospedale Maggiore Policlinico, University of Milan, Milan, Italy; 2 Laboratory “GA Maccacaro”, Department of Clinical Sciences and Community Health, University of Milan, Milan, Italy; 3 Microbiology Laboratory, Fondazione IRCCS Ca’ Granda Ospedale Maggiore Policlinico, Milan, Italy; Centre Hospitalier Universitaire Vaudois, FRANCE

## Abstract

**Objective:**

Presepsin (soluble CD14 subtype) has been shown to be beneficial as a sepsis marker in adults. Nevertheless, very few data are available in neonates. The aim of the present study was to determine reference ranges of presepsin in term and preterm neonates.

**Methods:**

Healthy term neonates and preterm neonates without clinical signs of infection admitted to the Neonatal Unit were consecutively enrolled. Presepsin concentrations in whole blood were measured using a point-of-care assay system located in the Unit. Demographic data, antenatal and perinatal variables commonly affecting C-reactive protein and procalcitonin values were considered.

**Results:**

Of the 684 neonates enrolled in the study, 484 (70.8%) were born at term and 200 (29.2%) were preterm (24–36 weeks’ gestation). In term infants, presepsin median value was 603.5 pg/mL (interquartile range: 466.5–791 pg/mL; 5th and 95th centiles: 315 and 1178 pg/mL respectively). In preterm infants, presepsin median value was slightly higher, equal to 620 pg/mL (interquartile range: 503–864 pg/mL; 5th and 95th centiles: 352 and 1370 pg/mL respectively). The reference ranges of presepsin we determined were much higher than those seen in healthy adults. No correlation between presepsin levels and postnatal age was observed, as well as no significant difference was demonstrated in preterm neonates at different gestational ages. None of the variables analyzed affected presepsin levels at a clinical significant extent.

**Conclusion:**

For the first time, this study provides reference ranges of presepsin in term and preterm neonates. Having reliable reference values is crucial for obtaining an adequate diagnostic accuracy. Based on our results, most variables commonly affecting C-reactive protein and procalcitonin values do not affect presepsin levels, which suggests that presepsin could be an effective sepsis marker. Further investigations in large groups of neonates with sepsis are needed to determine the diagnostic and prognostic value of this biomarker.

## Introduction

Sepsis is still a major cause of morbidity and mortality in neonates, especially in preterm infants [1]. Mortality rate can reach 60–70% in very low birth weight infants (birth weight < 1500 g) [2]. Beyond being a life-threatening condition, sepsis can cause sequelae in survivors and significantly impair neurodevelopmental outcome [3,4]. Because the disease can progress rapidly to septic shock and multiple organ dysfunction syndrome, early diagnosis is crucial to improve survival. Unfortunately, the diagnosis of sepsis may be difficult in the neonate. The earliest signs of the disease are often subtle and non specific, and can be easily confused with signs of non-infectious diseases [1].

Blood culture is the gold standard for the diagnosis of sepsis, but at least 48–72 hours are needed for the results and the number of false negative results is not negligible, particularly in early-onset sepsis, for which the blood culture sensitivity is reported to be less than 10% [5]. In the last decades, a great deal of efforts has been focused on biochemical markers. To date, C-reactive protein (CRP) and procalcitonin (PCT) are the most widely used markers for sepsis management in neonatal intensive care unit (NICU). CRP lacks the specificity to consistently discriminate between infections and non-infectious inflammatory conditions; furthermore, it rises late [6–10]. PCT has the advantage of increasing faster, and seems to be more specific for bacterial infections; however, its values depend on gestational age (GA) and postnatal age, particularly in the first days of life [8–11]. Although the reliability of these and other biomarkers raises when used together, the search for the optimal sepsis biomarker is far from being concluded.

In 2005, Yaegashi et al. [12] identified a new biomarker, named soluble CD14 subtype (sCD14-ST) or presepsin. Cluster of differentiation 14 (CD14) is a glycoprotein expressed on the membrane surface of various cells, such as monocytes, macrophages and granulocytes, and serves as a high-affinity receptor for complexes of lipopolysaccharides (LPSs) and LPS-binding proteins (LBPs), activating the toll-like receptor 4 (TLR4)-specific proinflammatory signaling cascade against infectious agents. After TLR4 activation, the complex LPS-LBP-CD14 is internalized into a phagolysosome. Soluble forms of CD14 are produced and released into circulation either by secretion following phagocytosis or through proteolytic cleavage on activated monocytes. Presepsin is a 13-kDa truncated form consisting of 64 amino acid residues [12–14].

In recent years, several authors evaluated the role of presepsin as a biomarker in adults. Based on the results of these studies, presepsin appears to offer some advantages over the most well known and widely used markers such as CRP and PCT, including an earlier increase in blood levels, higher sensitivity and specificity, and a prognostic value [12,15–23]. Furthermore, the cost of presepsin measurement is comparable to that of procalcitonin. Nevertheless, very few studies were conducted in pediatric population [24–28], and data on presepsin in large groups of term and preterm neonates have never been reported, neither for healthy babies nor for those with sepsis.

Therefore, we decided to perform a study to determine reference ranges of presepsin in healthy term neonates and in preterm neonates without clinical signs or symptoms of infection.

## Materials and Methods

### Study population

This was an observational single-center study, conducted at the Neonatal Unit of Fondazione IRCCS Ca’ Granda Ospedale Maggiore Policlinico of Milan, Italy, from February 2014 to August 2014. The study protocol was approved by the Ethics Committee of the Hospital. Written informed consent was obtained from parents for the inclusion in the study, and all procedures were in accordance with the Helsinki Declaration of 1975, as revised in 2008.

Healthy term neonates admitted to the nursery and preterm neonates without clinical signs or symptoms of local or systemic infection admitted to the NICU were consecutively enrolled. Exclusion criteria were the presence of major congenital malformations and lack of parental consent.

Term neonates were defined as those born at a GA greater than or equal to 37 and less than 42 weeks; preterm neonates were defined as those born at a GA less than 37 weeks. GA was established on the basis of best obstetric estimates, including last menstrual period and first or second trimester ultrasonography. Term neonates were defined as healthy when they appeared in good health conditions, with a normal physical examination and no need for medical management. Preterm neonates with a suspected sepsis were identified, according to Centers for Disease Control and Prevention (CDC) criteria [29], by the presence of one or more of the following clinical signs or symptoms with no other recognized cause and requiring antibiotic therapy on physician judgment: fever, hypothermia, grey/pale skin colour, hypotonia, hyporeactivity, apnea, polypnea, dyspnea, bradycardia, tachycardia, and hypotension. CDC criteria [29] were also used to identify infants with local infection. Small for gestational age (SGA) neonates were defined as those with a birth weight less than the 10^th^ centile for age, according to Bertino’s anthropometric charts [30].

### Study design

To determine presepsin levels, 100 μL of whole blood were obtained from term neonates via heel puncture on day 3–4 of life, at the time of Guthrie screening. The same amount of blood was obtained from preterm neonates via heel puncture or venipuncture between the 3^rd^ and the 7^th^ day of life, at the time of Guthrie screening or in conjunction with a blood sampling for clinical need. Blood was collected in sterile, endotoxin-free tube containing ethylenediaminetetraacetic acid (EDTA). All blood samples were stored at room temperature and processed within 4 hours after collection by physicians appropriately trained using a point-of-care assay system located in the NICU.

All neonates enrolled in the study were followed-up for one week after the blood sampling, and those presenting signs or symptoms of local or systemic infection were excluded from the analysis.

The time of blood sampling was decided after a preliminary study performed on a small group of 18 neonates with GA between 28 and 40 weeks of gestation assessed for presepsin 1, 3, 10, 21 days after birth: it emerged that neither postnatal age (p = 0.311), nor GA (p = 0.130), nor interaction age×GA (p = 0.129) appear to exert important effects on presepsin levels.

### Data collection

The following variables were considered in this study: mother’s information [fever during labor, maternal CRP at delivery, vaginal swab, premature (at least 1 hour before the onset of contractions)–rupture of the membranes (PROM), histological chorioamnionitis in preterm delivery, pre-eclampsia in preterm delivery], stained amniotic fluid, mode of delivery, birth place, gender, ethnicity, number of fetuses, GA, birth weight, weight for GA, Apgar score at 1 and 5 minutes, weight loss at the time of blood sampling, day at maximum weight loss, age at blood sampling, clinical course during the first 2 weeks of life. For preterm neonates, additional variables were considered: prenatal steroid exposure, need for resuscitation at birth, including oxygen and endotracheal intubation in delivery room, urinary output at the time of blood sampling, invasive and non-invasive ventilation, central venous catheterization, parenteral nutrition and antibiotic prophylaxis also at the time of sampling, surfactant administration and pharmacological treatment of patent ductus arteriosus (PDA) prior to sampling, CRP if performed and any surgery prior to sampling. The clinical risk index for babies (CRIB)-II score was also calculated for infants ≤ 32 weeks’ gestation.

### Presepsin measurement

Presepsin concentrations in whole blood were measured using a compact fully automated immunoassay analyzer, PATHFAST^TM^ System (LSI Medience Corporation, Japan / Mitsubishi Chemical Europe), based on a non-competitive chemiluminescence enzyme immunoassay [31]. The assay time is 15 minutes using a sample volume of 100 μL. Results are automatically corrected by entering the patient’s hematocrit value. For healthy term neonates, a value of 50% was used based on cord blood hematocrit determined for each newborn that ranged from 40 to 60%, mean value 50% as reported by Jopling et al. [32], considering that presepsin measurement is altered only when the difference between the effective value and the one entered is greater than 10%. For preterm neonates, hematocrit value was previously measured for routine test and entered for each of them.

### Statistical analysis

Presepin values in term and preterm neonates were modelled as a function of the variables listed above. To detect which among the above variables affects the presepsin values to a larger extent, separately for term and preterm neonates, the stepwise approach was adopted, having fixed a 0.30 significance level to entry a variable into the model and a 0.05 significance level to mantain a variable in the model. The presepsin values observed in the group of 18 neonates followed up to 21 days of postnatal life in the preliminary study were fitted with a repeated measures linear model: the effects of GA, age (linear component) and interaction “age (linear component) × GA” were estimated.

## Results

Of the 684 neonates enrolled in the study, 484 (70.8%) were born at term and 200 (29.2%) were preterm (24–36 weeks’ gestation). Five preterm infants were excluded from the analysis due to signs of infection in the week after the blood sampling.

Mean age at blood sampling was 3.6 days (SD 0.6) in term neonates and 3.9 days (SD 0.8) in preterm neonates. Mean presepsin value was 649 pg/mL (SD 257) in term neonates, and 720 pg/mL (SD 329) in preterm neonates.

In term infants, full records for all the variables under study were available. Among these variables possibly affecting presepsin levels, the following were excluded from further analysis: birth place (only 3 neonates were outborn), Apgar score at 5 min (all neonates had score above 7), and age at blood sampling (almost all neonates underwent blood sampling on the 3^rd^ or 4^th^ day of life). As shown in Table 1, presepsin levels of term neonates were little affected by the variables under study when singly considered. The stepwise selection analysis confirmed that only Apgar at 1 min was significantly associated with presepsin levels (p = 0.032) although to a negligible extent (R^2^ = 0.95%).

**Table 1:** Presepsin levels (pg/mL) in term neonates classified by the variables under study, singly considered (means ± standard errors).

variable	n_a_	mean_a_ ± SE_a_	n_b_	mean_b_ ± SE_b_	p
healthy_a_ *vs* pathological_b_ maternal status^1^	471	651 ± 11.9	13	606 ± 71.4	0.535
negative_a_ *vs* positive_b_ vaginal swab	454	649 ± 12.1	30	655 ± 47.0	0.907
normal_a_ *vs* stained_b_ amniotic fluid	447	655 ± 12.2	37	585 ± 42.2	0.116
spontaneous_a_ *vs* caesarean_b_ section	272	639 ± 15.6	212	663 ± 17.7	0.318
female_a_ *vs* male_b_	240	648 ± 16.6	244	651 ± 16.5	0.918
caucasian_a_ *vs* other_b_ ethnic groups	407	654 ± 12.8	77	625 ± 29.3	0.357
singleton_a_ *vs* twin_b_	467	653 ± 11.9	17	538 ± 62.2	0.068
gestational age 37-38_a_ *vs* 39-41_b_ weeks^2^	214	647 ± 17.6	270	651 ± 15.7	0.855
birth weight < 2500_a_ *vs* ≥ 2500_b_ g	25	686 ± 51.5	459	647 ± 12.0	0.464
AGA_a_ *vs* SGA_b_	429	651 ± 12.4	55	637 ± 34.7	0.712
Apgar at 1 min ≥ 8_a_ *vs* < 8_b_	462	645 ± 11.9	21	767 ± 55.9	0.032
weight loss at blood sampling ≤ 10%_a_ *vs* > 10%_b_	412	652 ± 12.7	72	635 ± 30.3	0.606
day at maximum weight loss ≤ 4_a_ *vs* > 4_b_	417	646 ± 12.6	67	673 ± 31.4	0.427

AGA: appropriate for gestational age; SGA: small for gestational age

^1^) fever during labor and/or elevated maternal C-reactive protein at delivery

^2^) completed weeks of gestation

In preterm infants, full records for all the variables under study were available, except for vaginal swab (168 missing cases due to preterm delivery). Among these variables, the following were excluded from further analysis: maternal health status, i.e. fever during labor and/or elevated maternal CRP at delivery (only 1 pathological case), vaginal swab (because of missing data), histological chorioamnionitis (only 8 positive cases), pre-eclampsia (only 7 cases), amniotic fluid (only 7 cases of stained fluid), birth place (only 16 outborn), urinary output at the time of blood sampling (only 1 case of diuresis < 1 ml/kg/hour), invasive ventilation at the time of blood sampling (only 6 cases), pharmacological treatment of PDA (only 3 cases treated prior to sampling), surgery (only 4 cases of surgery prior to sampling). As shown in Table 2, presepsin levels of preterm neonates were little affected by almost all the variables under study when singly considered. The stepwise selection analysis confirmed that presepsin levels were significantly higher in SGA neonates (p = 0.002, R^2^ = 5.1%).

**Table 2:** Presepsin levels (pg/mL) in preterm neonates classified by the variables under study, singly considered (means ± standard errors).

variable	n_a_	mean_a_ ± SE_a_	n_b_	mean_b_ ± SE_b_	p
prenatal steroids no_a_ *vs* yes_b_	89	750 ± 34.8	106	694 ± 31.9	0.237
rupture of membranes ≥ 1_a_ *vs* < 1_b_ hour	47	711 ± 48.1	148	723 ± 27.1	0.826
spontaneous_a_ *vs* caesarean_b_ section	42	699 ± 50.9	153	726 ± 26.7	0.642
female_a_ *vs* male_b_	100	693 ± 32.9	95	748 ± 33.7	0.248
caucasian_a_ *vs* other_b_ ethnic groups	164	711 ± 25.7	31	766 ± 59.1	0.394
singleton_a_ *vs* twin_b_	111	723 ± 31.3	84	716 ± 36.0	0.881
gestational age ≥ 32_a_ *vs* < 32_b_ weeks^1^	158	713 ± 26.2	37	747 ± 54.2	0.575
birth weight ≥ 1500_a_ *vs* < 1500_b_ g	159	714 ± 26.1	36	746 ± 54.9	0.594
AGA_a_ *vs* SGA_b_	161	686 ± 25.3	34	881 ± 55.1	0.002
Apgar at 1 min ≥ 8_a_ *vs* < 8_b_	131	733 ± 28.7	62	697 ± 41.8	0.482
Apgar at 5 min ≥ 8_a_ *vs* < 8_b_	176	715 ± 24.8	17	790 ± 79.7	0.372
resuscitation in delivery room no_a_ *vs* yes_b_ ^2^	134	732 ± 28.5	61	693 ± 42.2	0.443
oxygen in delivery room no_a_ *vs* yes_b_	156	729 ± 26.4	39	683 ± 52.7	0.440
endotracheal intubation in delivery room no_a_ *vs* yes_b_	180	710 ± 24.5	15	834 ± 84.8	0.164
CRIB-II score ≤ 4_a_ *vs* > 4_b_ ^3^	28	692 ± 62.5	23	772 ± 69.0	0.394
ventilation at blood sampling no_a_ *vs* yes_b_ ^4^	149	701 ± 26.9	46	781 ± 48.4	0.150
central venous catheter at blood sampling no_a_ *vs* yes_b_	143	702 ± 27.5	52	769 ± 45.6	0.205
parenteral nutrition at blood sampling no_a_ *vs* yes_b_	59	744 ± 42.9	136	709 ± 28.3	0.506
antibiotic prophylaxis at blood sampling no_a_ *vs* yes_b_	130	722 ± 28.9	65	716 ± 40.9	0.911
surfactant in the days before sampling no_a_ *vs* yes_b_	164	713 ± 25.7	31	754 ± 59.2	0.534
weight loss at blood sampling ≤ 10%_a_ *vs* > 10%_b_	152	721 ± 26.8	43	717 ± 50.3	0.944
day at maximum weight loss ≤ 4_a_ *vs* > 4_b_	96	720 ± 33.7	99	720 ± 33.2	0.995
day at blood sampling ≤ 4_a_ *vs* > 4_b_	139	691 ± 27.7	56	791 ± 43.7	0.055

AGA: appropriate for gestational age; SGA: small for gestational age

^1^) completed weeks of gestation

^2^) at least ventilation with mask

^3^) CRIB (clinical risk index for babies)-II score: calculated in infants ≤ 32 weeks’ gestation

^4^) all ventilated neonates were on non-invasive ventilation

Table 3 reports the distribution of prepsepsin levels in term neonates (with and without exclusion of cases with Apgar score < 8) and preterm neonates (with and without exclusion of SGA subjects). As expected on the basis of results previously reported (i.e. the very low values of R^2^), the exclusion of the term neonates with Apgar score < 8 does not affect the estimates of the centiles, whereas the exclusion of SGA preterm neonates decreases the 95^th^ centile by 8% only.

**Table 3:** Centiles of the distribution of presepsin levels (pg/mL) in term and preterm neonates.

Group	n	5^th^	10^th^	25^th^	50^th^	75^th^	90^th^	95^th^
term	484	**315**	371	466	604	791	1000	**1178**
term^1^	462	**311**	366	462	600	773	986	**1178**
preterm	195	**352**	390	503	620	864	1160	**1370**
preterm^2^	161	**352**	389	499	595	824	1049	**1256**

^1^) after exclusion of 21 neonates with Apgar score at 1 min < 8

^2^) after exclusion of 34 SGA neonates

## Discussion

Sepsis is a life-threatening condition and continues to be a major challenge for physicians, especially in intensive care units. Biomarkers play a pivotal role in early diagnosis, risk stratification, therapy monitoring and prognosis. In recent years, presepsin, a circulating molecule fragment derived from CD14, has been shown to be beneficial as sepsis marker in adults [12,15–23,33–36]. Nevertheless, very few data are available in neonates. Given that reliable reference values are crucial for obtaining an adequate diagnostic accuracy, we determined for the first time reference ranges of presepsin in a large group of neonates.

The preliminary study we conducted on a small group of neonates with different GA, who underwent repeated measurements in the first three weeks of life, made us confident enough that postnatal age does not affect presepsin levels, contrary to what occurs for CRP and PCT. That was in agreement with previous findings [26,27]. Therefore, we decided to withdraw blood sampling in the first week of life, at the same time of Guthrie screening.

Term and preterm neonates were evaluated separately, considering that preterm infants are often suffering from diseases related to prematurity and consequently not classifiable as “healthy”. In term infants, presepsin median value on day 3 or 4 of life was 603.5 pg/mL (interquartile range: 466.5–791 pg/mL; 5^th^ and 95^th^ centiles: 315 and 1178 pg/mL respectively). In preterm infants without clinical signs or symptoms of local or systemic infection, presepsin median value between the 3^rd^ and the 7^th^ day of life was slightly higher, equal to 620 pg/mL (interquartile range: 503–864 pg/mL; 5^th^ and 95^th^ centiles: 352 and 1370 pg/mL respectively). In 2012, Mussap et al. [24] reported a presepsin median value of 578 pg/mL (interquartile range: 453–796 pg/mL) in 26 non-septic preterm neonates with a GA between 26 and 36 weeks and a postnatal age between 25 and 160 hours. In the same year, Casani et al. [25] found a presepsin mean value of 953 pg/mL (interquartile range: 661–1114 pg/mL) in cord blood from 64 term or near-term (34–36 weeks’ gestation) neonates, and a mean value of 741 pg/mL (interquartile range: 490–937 pg/mL) in blood samples collected on the 3^rd^ day of life in 24 of the 64 infants enrolled. In a case-control study by Poggi et al. [26], conducted to evaluate the diagnostic accuracy of presepsin for the diagnosis of late-onset sepsis in preterm infants with GA ≤ 32 weeks and postnatal age between 4 and 60 days, presepsin median value at enrollment was 562 pg/mL (interquartile range: 337–726 pg/mL) in the 21 control subjects. In another recent case-control study by Topcuoglu et al. [27], a presepsin median value of 530 pg/mL (interquartile range: 363–580 pg/mL) was reported in a group of 40 control subjects with GA ≤ 32 weeks and postnatal age between 4 and 30 days. Very recently, Mussap et al. [28] reported a presepsin median value of 453 pg/mL (interquartile range: 309–526 pg/mL) in a group of 25 control subjects with GA less than 37 weeks.

The median value of presepsin we found in preterm neonates was slightly higher than that reported in the above studies, as well as the value corresponding to the 75^th^ centile. On the contrary, the values we found in term infants were lower compared to those reported by Casani [25] in his series of term/near-term neonates. None of the papers cited reported the 5^th^ and 95^th^ centiles.

In planning this study, we set ourselves two main objectives: 1) understanding whether presepsin levels change at different gestational ages; 2) evaluating the effect of antenatal and perinatal variables on presepsin values, considering that biomarkers such as CRP and PCT may be elevated in the first days of life or in non-infectious conditions [6–8,11].

First, we found presepsin levels slightly higher in preterm neonates compared to those born at term. In term neonates, no significant difference was found between early-term neonates (37–38 weeks’ gestation) and neonates with a GA more than 38 weeks, as well as in preterm neonates no significant difference was demonstrated between neonates with a GA less than 32 weeks (extreme-severe prematurity) and those with a GA of 32 weeks or more (moderate prematurity-near-term). For term neonates, no data are available in the literature to compare with our findings, while for premature babies our results are in agreement with those reported by Mussap et al. [24] that found no difference in the two subgroups of premature babies studied, with a GA of 30 weeks or less and a GA more than 30 weeks. Therefore, we suggest to adopt an unique reference range for term neonates and an unique reference range for those born preterm, regardless of GA.

Second, we tried to detect antenatal or perinatal variables, particularly infectious and pro-inflammatory risk factors, that could affect presepsin levels in non-infectious neonates. Identifying variables that influence the interpretation of a biomarker in symptomatic neonates or in neonates at risk for infection may represent an important aid in the differential diagnosis of sepsis. It is well known that an increased level of CRP, the most widely used marker of sepsis in NICU, is not necessarily diagnostic for sepsis, as elevations may also occur due to the physiologic rise after birth or non-infectious conditions, such as maternal fever during labor, PROM, vaginal delivery, fetal distress, perinatal asphyxia, meconium aspiration, respiratory distress syndrome, and surfactant administration [6–8]. In addition, Chiesa et al. [8] observed that neonates prenatally exposed to steroids had increased CRP concentrations. None of the variables listed above significantly affected presepsin levels in our study.

Among the variables analyzed in our study, only Apgar score at 1 min (≥ 8 *vs* < 8) and weight for GA (AGA *vs* SGA) appeared to be slightly, but significantly associated with presepsin levels. Nonetheless, the exclusion of term neonates with Apgar score at 1 min < 8 and of SGA preterm neonates resulted in a small decrease of the centiles above the median, so that these two variables can be safely ignored in the definition of the reference limits for presepsin in neonates.

In preterm neonates ≤ 32 weeks’ gestation, CRIB-II score was calculated within 1 hour after admission to the NICU [37]. A CRIB-II score > 4 is associated with a higher risk of mortality [38]. No difference in presepsin values was observed between CRIB-II score > 4 and ≤ 4. Finally, as increased presepsin concentrations were observed in adult patients with kidney disfunction, we also evaluated as factors possibly affecting presepsin values an excessive weight loss and an urinary output < 1 mL/kg/hour. No difference in presepsin values was observed between weight loss at blood sampling ≤ 10 and > 10%. Only one preterm neonate enrolled in the study had oliguria. Therefore, we believe that this aspect should be investigated in a population of selected infants [39–41].

Given that the variables analyzed in our study did not affect presepsin levels at a clinical significant extent, we are confident that presepsin might discriminate between infections and non-infectious inflammatory conditions better than the currently most used biomarkers.

The reference ranges of presepsin we determined, as well as those reported by other authors in infants without signs of infection, were substantially higher than those seen in healthy adults [12,15,18,20,21,40]. Very recently, Giavarina et al. [42] reported that the reference limits for presepsin in 200 adult subjects free from inflammatory diseases were 55–184 pg/mL, corresponding to the 2.5^th^ and 97.5^th^ centiles. The activation of the innate immune system occurring after birth as a result of the transition from the normally sterile intra-uterine environment to a world that is rich in foreign antigens could partly explain the higher levels of presepin seen in healthy babies compared to healthy adults. Following birth, the neonatal skin and gut are rapidly colonized with microbial flora and this is a continuous stimulus to the innate immune system [43–45]. Moreover, it is known that TLR sensor function is well developed in newborns and an excessive reactivity of the TLR innate response has also been described. In agreement with other authors, Levy et al. [45] demonstrated that preterm and full-term neonates express significantly greater levels of TLR4 on peripheral blood monocytes both at baseline and after LPS stimulation, compared with adults. In the same study, they also observed significantly greater CD14 expression at baseline and after LPS stimulation in full-term neonates compared with adults.

Although presepsin values found in healthy neonates are higher than in adults and show a greater variability, we expect that presepsin could be a reliable sepsis marker in neonates, as in adults, considering that concentration levels seem to be independent of GA, postnatal age and most variables commonly affecting CRP and PCT values. To date, only three studies [26–28] were conducted on small groups of neonates to evaluate the diagnostic accuracy of presepsin in the neonatal period. According to these studies, presepsin seems to be an accurate test for the diagnosis of late-onset sepsis in preterm infants.

The strength of our study includes the high number of term and preterm neonates enrolled, as well as the high number of variables studied. Strict inclusion criteria allowed us to determine reference ranges of presepsin in neonates without infectious diseases. Furthermore, presepsin measurement was always performed in the NICU by few physicians appropriately trained.

The main limitation of our study was that we have not evaluated a large group of extremely preterm infants.

In the present study, we have determined for the first time reference ranges of presepsin in a large group of term and preterm neonates. Presepsin values were found to be slightly higher in preterm neonates compared to those born at term, and higher than in healthy adult population. Based on our results, most variables commonly affecting CRP and PCT values do not affect presepsin levels, which suggests that presepsin could be an effective sepsis marker. Further investigations in large groups of neonates with sepsis are needed to determine the diagnostic and prognostic value of this biomarker.
